# Improving potency of *Chlamydia trachomatis* major outer membrane protein muli-epitope DNA vaccine by fusion with human papillomavirues 6b L1

**Published:** 2011-01-10

**Authors:** Wen Xu, Zhaohui Shi, Jun Chen, Ruifeng Meng, Wenci Gong, Shanli Zhu, Lifang Zhang

**Affiliations:** 1 Department of Microbiology and Immunology, Wenzhou Medical College, Wenzhou, Zhejiang 325035, PR China

## 

Effective adjuvants are needed to design effective vaccines against *Chlamydia trachomatis* (Ct). The aim of this study was to observe the immune response stimulated by inoculation of a DNA vaccine encoding a fusion protein comprising multiple Ct major outer membrane protein (MOMP) epitopes and human papillomavirus 6b L1 (HPV 6b L1) as the basis for designing a novel DNA vaccine against genital Chlamydia infections. The recombinant sequence encoding MOMP multi-epitopes was tandemly inserted and engaged downstream of HPV 6b L1 to construct a plasmid vaccine. COS-7 cells were transfected with pcDNA3.1(+)/Ct MOMP 168 encoding the Ct MOMP multi-epitope gene and co-expressed with the nucleic vaccine plasmid pcDNA3.1(+)/HPV 6b L1/Ct MOMP 168, which contains both the HPV 6b L1 and Ct MOMP multi-epitope genes. In addition, BALB/ c mice were inoculated intramuscularly (i.m.) with pcDNA3.1(+)/HPV 6b L1/Ct MOMP 168 or pcDNA3.1(+)/Ct MOMP 168. Serum IgG and secretory IgA (sIgA) in vaginal washes were then measured. The expression of HPV 6b L1/Ct MOMP multi-epitope was confirmed by western blotting, confocal microscopy and RT-PCR. Mice vaccinated with pcDNA3.1(+)/HPV 6b L1/Ct MOMP 168 had significantly higher IgG and sIgA antibody titers than pcDNA3.1(+)/Ct MOMP 16 controls. The results show that genetic fusion of the molecular adjuvant HPV 6b L1 to Ct MOMP 168 significantly increases the antigen-specific antibody response induced by the Ct MOMP 168 DNA vaccine.

